# Abnormal maternal echocardiographic findings in triplet pregnancies presenting with dyspnoea

**DOI:** 10.1007/s00508-016-0954-4

**Published:** 2016-02-25

**Authors:** Marie Elhenicky, Klaus Distelmaier, Mariella Mailath-Pokorny, Christof Worda, Martin Langer, Katharina Worda

**Affiliations:** 1 Department of Obstetrics and Feto-maternal Medicine, Medical University of Vienna, Währinger Gürtel 18–20, 1090 Vienna, Austria; 2 Department of Internal Medicine II, Division of Cardiology, Medical University of Vienna, Währinger Gürtel 18–20, 1090 Vienna, Austria

**Keywords:** Triplet pregnancy, Maternal echocardiography, Pro-B-type natriuretic peptide, Multiple pregnancy, Dyspnoea in pregnancy

## Abstract

**Objective:**

The objective of our study was to evaluate the prevalence of abnormal maternal echocardiographic findings in triplet pregnancies presenting with dyspnoea.

**Study design:**

Between 2003 and 2013, patients’ records of 96 triplet pregnancies at our department were analysed including maternal and fetal outcome, echocardiographic parameters and N-terminal pro-B-type natriuretic peptide (NT-proBNP) levels. After exclusion of triplet pregnancies with fetal demise before 23 + 0 weeks, selective feticide or missing outcome data, the study population consisted of 60 triplet pregnancies. All women with dyspnoea underwent echocardiography and measurement of NT-proBNP.

**Results:**

Dyspnoea towards the end of pregnancy was observed in 13.3 % (8/60) of all women with triplet pregnancies, and all of these women underwent echocardiography. The prevalence of abnormal echocardiographic findings in women with dyspnoea was 37.5 % (3/8) with peripartum cardiomyopathy in one woman. Median serum NT-proBNP was significantly higher in women with abnormal echocardiographic findings compared with those without (1779 ng/ml, range 1045–6076 ng/ml vs 172 ng/ml, range 50–311 ng/ml; *p* < 0.001 by Mann-Whitney-U Test).

**Conclusion:**

We conclude that triplet pregnancies presenting with dyspnoea show a high prevalence of abnormal echocardiographic findings. Since dyspnoea is a common sign in triplet pregnancies and is associated with a high rate of cardiac involvement, echocardiography and evaluation of maternal NT-proBNP could be considered to improve early diagnosis and perinatal management.

## Introduction

The prevalence of multiple pregnancies has increased over the past three decades due to increases in ovulation induction, in vitro fertilisation and childbearing at older ages [1, 2]. Triplet pregnancies are associated with significantly increased risks of maternal and fetal morbidity compared to singleton and twin pregnancies [3]. Despite advances in neonatal care, no significant improvement in the outcome of triplet pregnancies has been reported during the past three decades, and almost all triplets are born preterm before 34 weeks [4–6].

Women with triplet pregnancies have significantly higher frequencies of hypertension, pre-eclampsia and diabetes [7]. Furthermore, women carrying triplet pregnancies are under particular physical stress because already in the second trimester the uterus enlarges to a size comparable to that of a singleton at term; therefore many women with uncomplicated triplet pregnancy report physical discomfort including dyspnoea [8].

Symptoms of reduced cardiac function or heart failure may mimic normal physiological findings of late pregnancy, including persistent tiredness, oedema, orthopnoea and dyspnoea on exertion [9].

One cause of heart failure that affects women late in pregnancy or in the early puerperium is peripartum cardiomyopathy with a reported maternal mortality rate of 9 % [10]. It is defined as an idiopathic cardiomyopathy presenting with heart failure secondary to left ventricular systolic dysfunction at the end of pregnancy or in the months following delivery [9]. Risk factors of peripartum cardiomyopathy include non-Caucasian ethnicity, advanced maternal age, multiparity, poor socioeconomic status, prolonged tocolytic use (beta-adrenergic agonists), gestational hypertension, pre-eclampsia and multiple pregnancy [10–12].

N-terminal pro-B-type natriuretic peptide (NT-proBNP) is secreted from the cardiac ventricles in response to ventricular volume expansion and pressure overload [13–15]. Nowadays, it is a well-established marker of heart failure, being correlated with systolic and diastolic dysfunction, severity of cardiac failure and associated symptoms [16]. In 2009, Franz et al. described elevated NT-proBNP levels in healthy pregnant women compared to non-pregnant women [17], and in 2008 Forster et al. described elevated levels of NT-proBNP in women with peripartum cardiomyopathy [18].

To our knowledge, there are no data about the prevalence of peripartum cardiomyopathy or abnormal cardiac function in women with triplet pregnancies. Thus, this study aimed to evaluate the prevalence of abnormal echocardiographic findings as well as NT-proBNP levels in a large cohort of women with triplet pregnancies.

## Materials and methods

This retrospective cohort study aims to evaluate the prevalence of abnormal maternal echocardiographic findings in triplet pregnancies treated at our tertiary referral centre as part of routine antenatal care. Ethical approval was obtained from the Ethics Committee of the Medical University of Vienna, reference number 1633/2012.

During a study period of 10 years, over 26.000 pregnancies were referred to our hospital. Of those, 96 women (0.38 %) presented with a triplet pregnancy. Medical records of all women with triplet pregnancies who attended our department between December 2003 and December 2013 were reviewed. Demographic characteristics and data on pregnancy outcome were collected from the hospital maternity records.

Of all triplet pregnancies, 14.6 % (14/96) were excluded because of fetal demise before 23 + 0 weeks, and 20.8 % (20/96) were excluded because of selective feticide (elective determination). Furthermore, 2.1 % (2/96) were excluded because of missing outcome data. Finally, the study population consisted of 60 women with triplet pregnancies.

Women with triplet pregnancies routinely underwent ultrasound screening at 11 + 0 to 13 + 6 weeks since first trimester screening has been established. Chorionicity and amnionicity were routinely determined at first trimester screening by ultrasound examination of the dividing membranes characteristics and by examination of the placenta after birth. At 20–22 weeks, pregnant women routinely underwent anatomy scan. From 16 weeks of gestation onward, triplet pregnancies were routinely seen in 2-weekly intervals at our institution’s outpatient clinic. All women were seen by operators specialised in the field of multiple pregnancies. If a woman presented with dyspnoea (which was clinically diagnosed by an experienced consultant), clinical assessment using 2-dimensional transthoracic and Doppler echocardiography was performed.

All patients’ medical records were reviewed for maternal parameters such as maternal age, body mass index (BMI), pre-existing cardiac disease, smoking behaviour, parity, method of conception, pregnancy complications (e.g. hypertension, pre-eclampsia or diabetes), use of tocolytics, mode of delivery and neonatal outcome parameters such as live birth, gestational age at delivery, birth weight, apgar 5 min after birth, arterial cord blood pH and admission to the neonatal intensive care unit.

Furthermore, all patients’ medical records were reviewed for reports of dyspnoea, maternal echocardiographic ultrasound examinations and maternal serum NT-proBNP levels.

The main outcome parameter of this study was the prevalence of abnormal maternal echocardiographic findings in women with triplet pregnancies.

Furthermore, we evaluated the prevalence of peripartum cardiomyopathy. We used the definition of the European Society of Cardiology Study Group on Peripartum Cardiomyopathy for defining peripartum cardiomyopathy: ‘Peripartum cardiomyopathy is an idiopathic cardiomyopathy presenting with heart failure secondary to left ventricular systolic dysfunction towards the end of pregnancy or in the months following delivery, where no other cause of heart failure is found. It is a diagnosis of exclusion. The left ventricle may not be dilated but the ejection fraction is nearly always reduced below 45 %’ [9].

Echocardiographic results were analysed according to the guidelines of the National Heart, Lung and Blood Institute and Office of Rare Diseases (National Institutes of Health) Workshop on Peripartum Cardiomyopathy including an ejection fraction of 45 % or less, fractional shortening of less than 30 %, or both and end-diastolic dimension of greater than 2.7 cm/m^2^ body surface area [19].

Statistical analyses were performed with SPSS software (version 18.0; SPSS, Chicago, IL). Parametric continuous variables were summarised as means (± standard deviation), non-parametric continuous variables as medians (minimum and maximum) and categorical data as percentages. Categorical variables were analysed using Fisher’s exact test, continuous variables were compared using unpaired T-test, Kruskal-Wallis or Mann-Whitney U-test. For infant outcomes, the correlation within sets of triplets was accounted for by considering robust estimates of variance based on the method of generalised estimating equations (GEE) [20].

## Results

Sixty triplet pregnancies were analysed, including 63.3 % (38/60) trichorionic and 36.7 % (22/60) dichorionic triplet pregnancies. Patients’ characteristics are shown in Table 1.

**Table 1:** Patients’ characteristics

	Triplet pregnancies without abnormal maternal echocardiographic findings (*n* = 57)	Triplet pregnancies with abnormal maternal echocardiographic findings (*n* = 3)
Maternal age (years; mean, SD)	30.8 (± 5.0)	31.0 (± 3.8)
BMI (kg/m^2^; mean, SD)	24.0 (± 3.5)	25.2 (± 1.1)
Pre-existing cardiac disease (*n*, %)	1 (1.8 %)	–
Smoking (*n*, %)	10 (17.9 %)	–
Nulliparity (*n*, %)	41 (71.9 %)	3 (100 %)
Method of conception (*n*, %)		
- ART	52 (100 %)^a^	3 (100 %)^a^
Diabetes mellitus (*n*, %)	13 (22.9 %)	1 (33 %)
Hypertension (*n*, %)	9 (15.8 %)	–
Pre-eclampsia (*n*, %)	3 (5.3 %)	1 (33 %)
HELLP syndrome (*n*, %)	1 (1.7 %)	–
Use of tocolytics (*n*, %)	47 (82.5 %)	3 (100 %)
Mode of delivery (*n*, %)		
- Caesarean section	56(98.2 %)	3 (100 %)
- Vaginal delivery	1 (1.8 %)	–
Dyspnoea (*n*, %)	5 (8.8 %)^b^	3 (100 %)^b^
GA (wks) at echocardiography (median, range)	31.9 (27.0–33.3)	31.9 (30.3–33.6)

*SD* standard deviation, *BMI* body mass index, *ART* assisted reproductive technique, *GA* gestational age, *wks* weeks, *n* number

^a^In five cases no data about method of conception were available

^b^ *p* = 0.002 by Fisher’s exact test

The prevalence of abnormal echocardiographic findings in women with dyspnoea was 37,5 % (3/8), with peripartum cardiomyopathy in one woman. The whole study population showed a high rate of diabetes (23.3 %; 14/60) and hypertension (15 %; 9/60). One of the three women with abnormal echocardiographic findings had diabetes and one had pre-eclampsia.

Dyspnoea was observed in 13.3 % (8/60) of all women with triplet pregnancies, and all of these women underwent clinical assessment using 2-dimensional transthoracic and Doppler echocardiography. Mean gestational age at examination was 27.8 (± 4.6) weeks of gestation. Three women had abnormal echocardiographic findings (abnormal left ventricular function, pericardial effusion). All other women with dyspnoea had normal echocardiographic results, and in one woman the previously described mitral insufficiency was confirmed without worsening.

Women with abnormal echocardiographic findings are described in detail:

Patient no. 1 was a 30-year-old woman with a dichorionic triamniotic triplet pregnancy and mild pre-eclampsia from 26 weeks of gestation onwards. As antihypertensive therapy she received methyldopa. At 30.3 weeks of gestation, she presented with dyspnoea and oedema. Echocardiography showed normal left ventricular ejection fraction, impaired cardiac relaxation and pericardial effusion, and NT-proBNP was 1045 ng/ml. Maternal infections were excluded. The next day, dyspnoea and oedema worsened, additionally the woman reported palpitations, and therefore caesarean section was performed. Clinical improvement began right after delivery, and after 6 days the mother was free of symptoms and discharged from hospital.

Patient no. 2 was a 28-year-old woman with a trichorionic triamniotic triplet pregnancy who presented with dyspnoea at 33.6 weeks of gestation. Echocardiography showed abnormal left ventricular diastolic function and impaired cardiac relaxation, and NT-proBNP was 1779 ng/ml. The next day the woman reported worsened dyspnoea, and caesarean section was performed. She recovered soon after delivery, and NT-proBNP decreased to 372 ng/ml. Five days after delivery the mother was free of symptoms and discharged from hospital.

Patient no. 3 was a 37-year-old woman with a trichorionic triamniotic triplet pregnancy with insulin-dependent gestational diabetes. She presented with dyspnoea at 31.9 weeks of gestation. Echocardiography showed a left ventricular ejection fraction of 50 %. One day later, caesarean section was performed due to more pronounced dyspnoea resulting in marked limitation of physical activity in accordance with New York Heart Association functional class III (The Criteria Committee of the NYHA, 1994). After delivery, the patient was transferred to the Department of Cardiology, and echocardiography showed a reduced left ventricular ejection fraction of 36 % and pericardial effusion, and the diagnosis of peripartum cardiomyopathy was made. NT-proBNP was 6076 ng/ml. Therapy with ramipril and bisoprolol was started, and the symptoms got better. Twenty-four days after delivery, echocardiography showed a left ventricular ejection fraction of 50 %, abnormal septal movements and mitral insufficiency. The patient felt much better and could be discharged. In the following months she had several check-ups with mild or no symptoms of heart failure reported.

NT-proBNP levels were measured in all women with dyspnoea (13.3 %, 8/60) at the time of maternal echocardiography. Median NT-proBNP levels were significantly higher in women with abnormal echocardiographic findings compared with those without (1779 ng/ml, range 1045–6076 ng/ml vs 172 ng/ml, range 50–311 ng/ml; *p* < 0.001 by Mann-Whitney-U Test).

Neonatal outcome is shown in Table 2. Neonatal outcome did not significantly differ between women with abnormal echocardiographic findings and those without.

**Table 2:** Neonatal outcome

	Triplets without abnormal maternal echocardiographic findings (*n* = 171)	Triplets with abnormal maternal echocardiographic findings (*n* = 9)
Live births	167 (97.7 %)	9 (100 %)
GA at delivery (weeks; median, range)	32.7 (23.3–35.7)	32.0 (30.4–34.0)
Apgar 5 min after delivery (median, range)	9 (1–10)	9 (7–10)
Arterial cord blood pH (median, range)	7.30 (6.95–7.40)	7.10 (7.00-7.28)
Birth weight (g; median, range)	1567 (361–2759)	1750 (1280–2400)
NICU (*n*, %)	157 (94 %)	9 (100 %)
SGA (*n*, %)	17 (10.1 %)	0

*GA* gestational age, *SD* standard deviation, *NICU* neonatal intensive care unit, *SGA* small for gestational age (< 5th percentile), *n* number

## Comment

In our study, we evaluated the prevalence of abnormal maternal echocardiographic findings in triplet pregnancies. We could present a high prevalence of abnormal echocardiographic findings in women with dyspnoea (37.5 %). In one of these (1/3), the diagnosis of peripartum cardiomyopathy was made. Abnormal echocardiographic findings were associated with diabetes and pre-eclampsia. All women with abnormal echocardiographic findings had markedly elevated NT-proBNP levels.

This is one of the largest studies evaluating the prevalence of abnormal echocardiographic findings in triplet pregnancies. All pregnant women with dyspnoea underwent echocardiography. As typical for a retrospective study design, our report shows potential limitations. Since we did not perform echocardiography in all triplet pregnancies, we could not exclude that even women with shortness of breath which seems appropriate for pregnancy might have reduced cardiac function. Nevertheless, in those who presented with more pronounced symptoms, the percentage of abnormal echocardiographic findings was very high. Furthermore NT-proBNP levels were not measured longitudinally and not in all women with triplet pregnancies; therefore we cannot present normal values for NT-proBNP in triplet pregnancies.

The high rate of dyspnoea (13.3 %) in triplet pregnancies could be explained by haemodynamic factors—even in singleton pregnancies there is a significant increase in blood volume and cardiac output resulting in transient left ventricular remodelling and hypertrophy [21–23]. This increase may be more pronounced in triplet pregnancies and could further lead to higher haemodynamic stress and reduced cardiac function with abnormal echocardiographic findings in some triplet pregnancies.

Hypertension and pre-eclampsia have been described as risk factors for peripartum cardiomyopathy [10, 11]. We observed high rates of hypertension (15 %; 9/60) and pre-eclampsia (6.7 %; 4/60) in our population of triplet pregnancies, which is in accordance to previously reported data [7]. The high prevalence of abnormal echocardiographic findings in our population could partially be triggered by the high rate of these complications.

In our study, abnormal echocardiographic findings were observed after 30 weeks of gestation. Since triplet pregnancies were delivered at a median gestational age of 32–34 weeks, reduced cardiac function occurred in the last month of pregnancy in our study population, which is in accordance with the time of diagnosis of peripartum cardiomyopathy in singletons [4, 6].

We showed a prevalence of peripartum cardiomyopathy of 1.7 % in triplet pregnancies, which is higher than previously reported prevalence of 0.1–0.3 % in different populations including mainly singleton pregnancies [24, 25]. It has been suggested that an abnormal maternal immunologic response to fetal antigen could lead to peripartum cardiomyopathy [26]. One could assume that the amount of fetal antigen is higher in triplet pregnancies, which could lead to a higher prevalence of peripartum cardiomyopathy. However, available data regarding abnormal immunological response in the aetiology of peripartum cardiomyopathy are conflicting [18, 26, 27, 28].

It has been described that NT-proBNP, which is a quantitative serum marker of cardiac disease reflecting systolic and diastolic ventricular dysfunction, is elevated in patients with heart failure and in women with peripartum cardiomyopathy [29, 30]. Forster et al. described elevated levels of NT-proBNP in women with peripartum cardiomyopathy [18]. We measured significantly elevated levels of NT-proBNP in all women with abnormal echocardiographic findings, with no overlap between measurements of women with and without abnormal echocardiographic findings.

Although only one woman with abnormal echocardiographic findings fulfilled the diagnostic criteria of peripartum cardiomyopathy, one could assume that also the other women with abnormal echocardiographic findings may have similar pathophysiological processes since some shared risk factors for peripartum cardiomyopathy, and all women with abnormal echocardiographic findings had significantly elevated levels of NT-proBNP. However, this remains speculative.

Since reduced cardiac function can mimic normal physiological findings of late pregnancy (dyspnoea, exhaustion), and these symptoms are even more pronounced in triplet pregnancies, diagnosis of reduced cardiac function could be challenging. Nevertheless, 37.5 % (3/8) of all women who presented with remarkable dyspnoea had abnormal echocardiographic findings, and 62.5 % (5/8) had normal echocardiographic results.

Triplet pregnancies presenting with dyspnoea show a high prevalence of abnormal echocardiographic findings. Since dyspnoea is a common sign in triplet pregnancies and is associated with a high rate of cardiac involvement, echocardiography and evaluation of maternal NT-proBNP could be considered to improve early diagnosis and perinatal management.

### Financial support

This research received no specific grant from any funding agency, commercial or not-for-profit sectors.
